# Effects of Active Conductance Distribution over Dendrites on the Synaptic Integration in an Identified Nonspiking Interneuron

**DOI:** 10.1371/journal.pone.0002217

**Published:** 2008-05-21

**Authors:** Akira Takashima, Masakazu Takahata

**Affiliations:** Department of Biological Sciences, Faculty of Science, Hokkaido University, Sapporo, Japan; Freie Universitaet Berlin, Germany

## Abstract

The synaptic integration in individual central neuron is critically affected by how active conductances are distributed over dendrites. It has been well known that the dendrites of central neurons are richly endowed with voltage- and ligand-regulated ion conductances. Nonspiking interneurons (NSIs), almost exclusively characteristic to arthropod central nervous systems, do not generate action potentials and hence lack voltage-regulated sodium channels, yet having a variety of voltage-regulated potassium conductances on their dendritic membrane including the one similar to the delayed-rectifier type potassium conductance. It remains unknown, however, how the active conductances are distributed over dendrites and how the synaptic integration is affected by those conductances in NSIs and other invertebrate neurons where the cell body is not included in the signal pathway from input synapses to output sites. In the present study, we quantitatively investigated the functional significance of active conductance distribution pattern in the spatio-temporal spread of synaptic potentials over dendrites of an identified NSI in the crayfish central nervous system by computer simulation. We systematically changed the distribution pattern of active conductances in the neuron's multicompartment model and examined how the synaptic potential waveform was affected by each distribution pattern. It was revealed that specific patterns of nonuniform distribution of potassium conductances were consistent, while other patterns were not, with the waveform of compound synaptic potentials recorded physiologically in the major input-output pathway of the cell, suggesting that the possibility of nonuniform distribution of potassium conductances over the dendrite cannot be excluded as well as the possibility of uniform distribution. Local synaptic circuits involving input and output synapses on the same branch or on the same side were found to be potentially affected under the condition of nonuniform distribution while operation of the major input-output pathway from the soma side to the one on the opposite side remained the same under both conditions of uniform and nonuniform distribution of potassium conductances over the NSI dendrite.

## Introduction

Many nerve cells are endowed with a variety of voltage- and ligand-regulated conductances on their dendrites. Although synaptic inputs to each individual cell are primarily processed on the electrotonic basis of dendritic structure [1], their integration is critically affected by dynamic functions of those active conductances as well [2]. An important aspect of their functional significance in synaptic integration is how they are distributed over the dendrite: Are they distributed uniformly over dendrites or concentrated in specific neuronal region? It is also unknown for many neurons how the distribution pattern of active conductances affects processing of synaptic inputs on the dendrite.

Nonuniform distribution of active conductances over dendrites has been known in some specific neurons. It has been reported, e.g., in Purkinje cells of the vertebrate cerebellum that voltage-regulated inactivating as well as non-inactivating sodium conductances and others are distributed over the soma membrane whereas voltage-regulated calcium and potassium conductances as well as a calcium-regulated potassium conductance are exclusively distributed over dendrites [3]. The voltage-regulated calcium conductances are localized in discontinuously dispersed regions on dendrites to form ‘hot spots’ [4] (Llinás and Nicholson, 1971) for boosting synaptic input toward the cell body [5], [6]. It has been also reported in the hippocampal pyramidal cell that the distribution of voltage-regulated conductances are varied among the soma and dendrites [7]. A possibility that the high density of A-type potassium channels may counteract EPSP boosting provided by subthreshold sodium channel activation has been implicated by simulation on the dynamics of those conductances. It is important to note here that the distribution of active conductances over dendrites depends on the cell identity and that its physiological significance varies with the cell according to the functional role of the cell in behavioral control.

Nonspiking interneurons are found in the central nervous system of invertebrate, above all, arthropod animals. Those interneurons exert graded and continuous output on postsynaptic cells without generating spikes. They are involved in the neuronal circuit for sensory information processing [8], [9] and motor control systems [10], [11] in arthropods. The most intensively studied among them is the LDS (local directionally sensitive) interneuron that is identifiable in the terminal abdominal ganglion of crayfish, mediating lateral inhibition in the ascending mechanosensory system [12], [13]. It receives mechanosensory input from the tailfan monosynaptically by nicotinic-like acetylcholine receptors on the soma side [14] and transmits the evoked synaptic potential to the dendritic branches on the opposite side exerting inhibitory output to ascending projection interneurons [12], [13]. Three kinds of voltage-regulated potassium conductances have been quantitatively characterized by single-electrode voltage-clamp experiments [15] that, together with the three-dimensional morphometry of dendritic structure [16], enabled us to compose a multicompartment model of the LDS interneuron for simulating synaptic activities on its dendrite [17]. It remains unknown, however, how the potassium conductances are distributed over dendrites of the interneuron as well as how the synaptic activity is affected by specific distribution patterns.

In the present study, we systematically changed the distribution pattern of active conductances in the interneuron's multicompartment model and examined how the synaptic potential waveform was affected by each distribution pattern. The distribution pattern of active conductances in the model can be changed either manually [2], [18], [19] or automatically using genetic algorithms [20] or evolution strategy [21]. In this study, we hand-tuned the active conductance distribution systematically in six patterns. The effects of those distribution patterns on the spatio-temporal spread of synaptic potentials were compared with the uniformly distributed pattern as the target model. Functional significances of nonuniform distribution of active conductances over dendrites in the synaptic integration of the LDS interneuron will be discussed.

## Results

**Figure 1:**
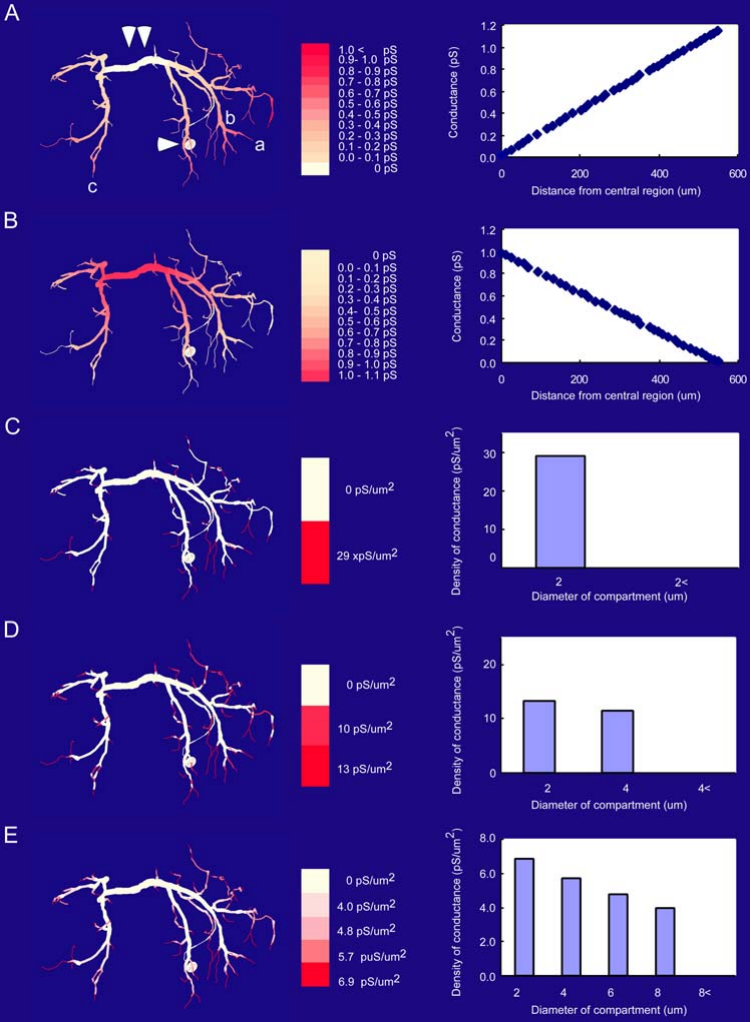
Distribution patterns of the delayed-rectifier type potassium conductance over dendrites of the LDS interneuron. Dendritic portions colored in deeper red (left) indicate distribution of active conductances in higher densities (middle). A: Increasing distribution of active conductances as the function of distance from the transverse segment (pattern II). B: Decreasing distribution of active conductances as the function of distance from the transverse segment (pattern III). C: Weighted distribution of active conductances over dendrites having a diameter smaller than 2 µm (pattern IV). D: Weighted distribution of active conductances over dendrites having a diameter smaller than 4 µm (pattern V). E: Weighted distribution of active conductances over dendrites having a diameter smaller than 8 µm (pattern VI). On the left is shown each distribution over the dendrite while the conductance value (A, B) and density (C, D, E) as a function of dendritic distance and diameter respectively are shown on the right. Those patterns also applied to other two types of potassium conductances. In all cases, the total amount of conductances was the same as the membrane conductance obtained in the voltage-clamp experiment.

Since the cell body of the LDS interneuron (shown with a single arrowhead in Fig. 1A) is not involved in the signal pathway from input synapses to output sites in the dendritic branches or in the axon initiating region, we first assumed no conductance on the soma membrane in all six cases. Input synapses have been reported to be located on fine processes with the diameter less than 8 µm µm on the soma side whereas output synapses are on the processes on the opposite side [22]. The synaptic input is summed up in the thick transverse segment on the midline (shown with a double arrowhead in Fig. 1A) and further transmitted to the output synapses [17]. Thus the transverse segment of the interneuron, having so large a diameter as to make it isopotential over its full length [16], serves as the integrating site for synaptic inputs just as the cell bodies of vertebrate neurons do. We therefore regarded this segment as the reference point on which basis the distributions of active conductances were systematically changed in the present simulation study.

### Effects of nonuniform distribution of active conductances on unitary synaptic activities and voltage responses to intracellular current injection

**Figure 2:**
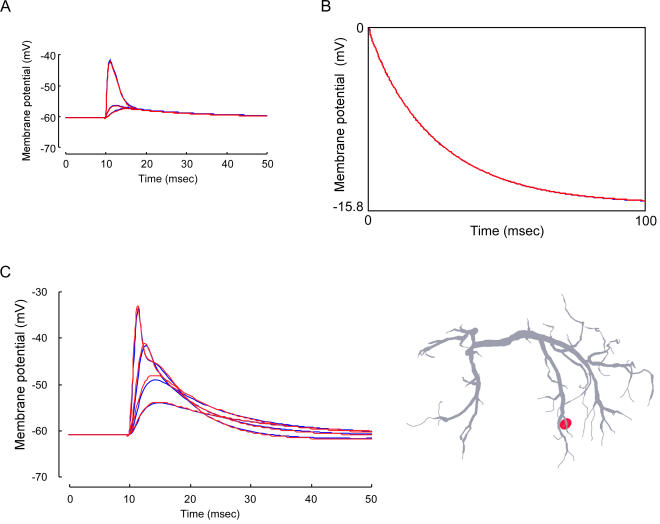
Effects of uniform (blue) and nonuniform (red) distribution of potassium conductances on the synaptic potential waveform and voltage response to current injection. A: Dendritic spread of a unitary synaptic potential. The time course of synaptic potentials in three representative regions (a, b and c shown in Fig. 1) is illustrated for the nonuniform distribution pattern II. B: Voltage responses to a step current injection in the midline region in a model having the nonuniform distribution pattern IV. The calculated unitary synaptic potentials for other nonuniform distribution patterns were closer to those calculated for the uniform distribution than those shown here while the calculated voltage responses for other distribution patterns were all the same with those shown here. Note that only red lines can be recognized in both panels where blue and red lines are superimposed. C: Effects of soma conductances on the spatio-temporal distribution of compound synaptic potentials over dendrites. The cell body was distributed by additional 40% of the whole active conductances that were uniformly distributed over dendrites. On the left are superimposed the waveforms of compound synaptic potentials recorded in the transverse segment and evoked by stimuli of different intensities that were mimicked by different number of activated synapses in the present simulation. The synaptic potential without soma conductance (red) is compared with that with the conductance (blue). The responses calculated for additional 10% and 20% soma conductances were all the same as those shown here.

We tested six patterns of active conductance distribution including the uniform distribution of conductances over dendrites (pattern I; see Materials and Methods). Different distribution patterns of voltage-regulated potassium conductances over the dendrites of the LDS interneuron had little effect on the spatio-temporal spread of a unitary EPSP caused by activation of any single synaptic terminal compartment on the soma side. The nonuniform distribution that yielded most remarkable difference from the uniform distribution (Fig. 2A) was the pattern II in Fig. 1. For three calculated regions including the synaptic site (a in Fig. 1A), a point in the dendrite on the soma side (b) leading to the transverse segment and an output site assumed on a contralateral dendrite (c), the membrane potential changes associated with a unitary synaptic activation are compared between the uniformly (blue) and nonuniformly (red) distributed active conductances (Fig. 2A). In this distribution, the active conductance of a compartment increased linearly as it is located farther from the thick transverse segment that bore no active conductance (Fig. 1A). For other nonuniform distribution patterns, the synaptic responses were closer to those calculated for the uniform distribution. It is noted here that even in the case of increasingly graded distribution of active conductances over dendrites (Fig. 2A), the difference was physiologically of no significance suggesting that the nonuniform distribution of active conductances in any pattern would have no substantial effect on the spatio-temporal spread of unitary synaptic potential over dendrites of the LDS interneuron, although the membrane potential change due to a single synaptic action at the synaptic site well exceeded the activation threshold for the conductances [15].

The voltage responses of the interneuron to intracellularly injected current were also found to be hardly affected by any pattern of nonuniform distribution of active conductances (Fig. 2B). The current was injected into the cell in the midline region and the voltage response to the injected current was recorded in the same region using a single electrode (see Materials and Methods). In the calculation shown in Fig. 2B, the active conductances were nonuniformly distributed in the pattern II. Similar results were obtained when they were distributed in other nonuniform patterns.

### Effects of active conductances in the soma membrane on synaptic activities

Invertebrate central neurons typically possess a cell body connected to dendrites with a thin process called primary neurite that is so fine and long as to separate itself electrotonically from the other parts of the cell. A possibility still remains, however, that the cell body has active conductances to exert functional effects on the synaptic integration in the dendrite. Assuming that voltage-regulated potassium conductances are also present, although partly, we investigated the effect of active soma membrane on the spatio-temporal spread of the compound synaptic potential over dendrites for three distribution patterns (see Materials and Methods). Regardless of the weighting factor for the conductance distribution on the soma membrane (10, 20, 40%), the spatio-temporal spread of the compound synaptic potential was hardly affected by distribution of active conductances over the somatic membrane (Fig. 2C). The dendritic membrane was uniformly distributed by active conductances throughout the simulation shown in Fig. 2C (see Materials and Methods). Although not shown in the figure, we confirmed that the active soma membrane had no functional significance in the spatio-temporal distribution of the compound synaptic potential even if the active conductances were distributed nonuniformly on the dendritic membrane.

The only difference observed in the experiment shown in Fig. 2C was that the medium-sized compound response was larger when the soma membrane was active than when it was passive regardless of the soma conductance weighting factor. The time course was not different in any response. It is true that the soma membrane has a significant effect on the calculated voltage response to intracellularly injected current in the transverse segment as can be seen in the fact that the soma membrane area, as well as the dendritic membrane area, has to be modified to make the model realistic (see Materials and Methods), but the result of our simulation suggests that the somatic conductance has almost no significant effect on the spatio-temporal spread of the synaptic potential over dendrites in the LDS interneuron.

### Effects of nonuniform distribution of active conductances on compound synaptic activities

**Figure 3:**
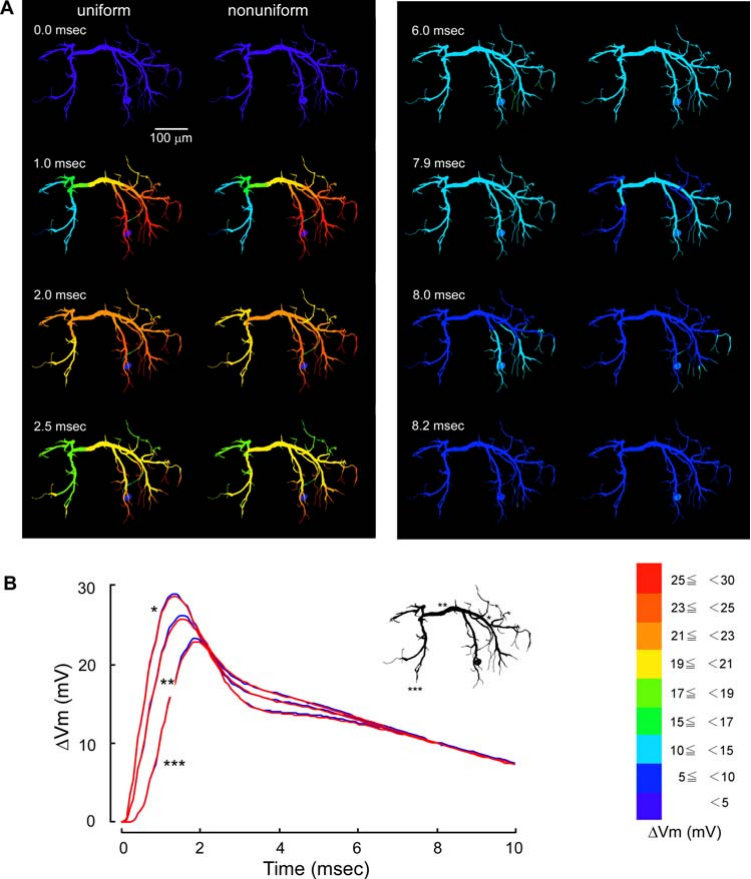
Effects of non-uniform distribution of active conductances in the pattern III on the dendritic spread of a compound synaptic potential. A: Spatial distribution of the compound synaptic potential in response to maximal sensory stimulation in a uniform (left) and a non-uniform model (right) where active conductances were distributed decreasingly from thick transverse segment to distal dendritic terminals except soma. B: Temporal distribution of the compound synaptic potential on the uniform (blue) and nonuniform (red) dendrite, calculated at a thick segment of the ipsilateral dendrite to the soma (*), at the transverse segment (**) and at a terminal of the contralateral dendrite (***).

**Figure 4 pone-0002217-g004:**
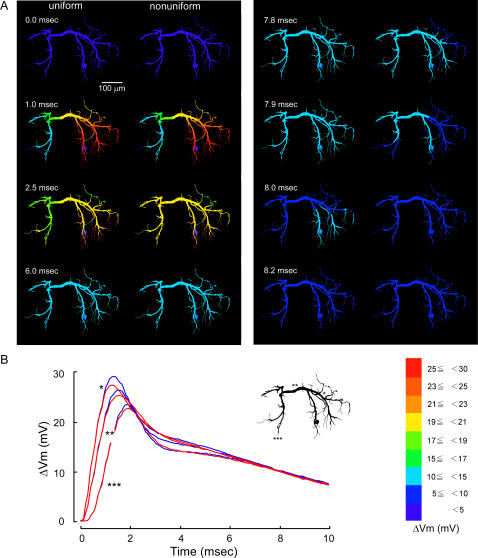
Effects of non-uniform distribution of active conductances in the pattern VI on the dendritic spread of a compound synaptic potential. Colors and symbols are the same as in Fig. 3. The active conductances were distributed only on dendrites with the diameter of less than 8 µm except soma. The synaptic potential waveform was slightly different from that calculated for the uniform distribution on the side ipsilateral to the synaptic input, but not different on the midline and on the opposite side.

The different distribution patterns of voltage-regulated potassium conductances over dendrites could have noticeable effects on the compound synaptic potential that was produced by spatial summation of unitary synaptic responses each of which was hardly affected by the presence nor distribution pattern of active conductances. In the following section, we describe the effects of nonuniform distribution of active conductances on the compound synaptic potential in increasing order of effectiveness. First, when they were distributed decreasingly from the thick transverse segment to distal branches (pattern III, Fig. 1B), the spatio-temporal spread of the compound synaptic potential showed little difference between uniform and nonuniform distribution (Fig. 3A). A difference can be seen in the spatial distribution of the synaptic potential around 2.0 and 8.0 msec after the onset of the synaptic potential, but it would be safe to conclude that there is no physiologically significant difference between them by examining the time course of those synaptic potentials in three representative regions, i.e., the ipsilateral dendrite, the transverse segment, and the contralateral dendrite (Fig. 3B). Second, when the active conductances were distributed only on dendrites having a diameter smaller than 8 µm (pattern VI, Fig. 1E), the peak time became slightly shorter with the smaller peak amplitude in a thick dendritic region on the way from the input terminal to the transverse segment (Fig. 4A) whereas both the peak time and amplitude were almost the same in the transverse segment and an output terminal in the dendrite on the side opposite to the synaptic input (Fig. 4B). Throughout the decay phase, the synaptic potential decreased faster in fine dendritic branches on the input side than other regions including the transverse segment and contralateral dendrites where the potential decreased simultaneously (Fig. 4A right column 7.8 ms–7.9 ms). In case the active conductances were distributed uniformly over dendrites, the synaptic potential decayed faster on the contralateral side than on the side ipsilateral to the soma (Fig. 4A left 8.0 ms).

**Figure 5 pone-0002217-g005:**
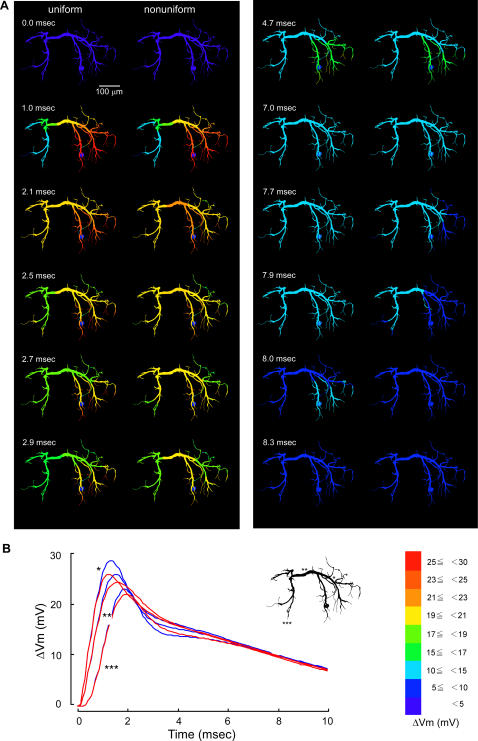
Effects of non-uniform distribution of active conductances in the pattern II on the dendritic spread of a compound synaptic potential. Colors and symbols are the same as in Fig. 3. The active conductances were distributed increasingly from the thick transverse segment to distal dendritic terminals except soma. The synaptic potential waveforms were different from those calculated for the uniform distribution not only on the side ipsilateral to the synaptic input, but also on the midline and on the opposite side.

**Figure 6 pone-0002217-g006:**
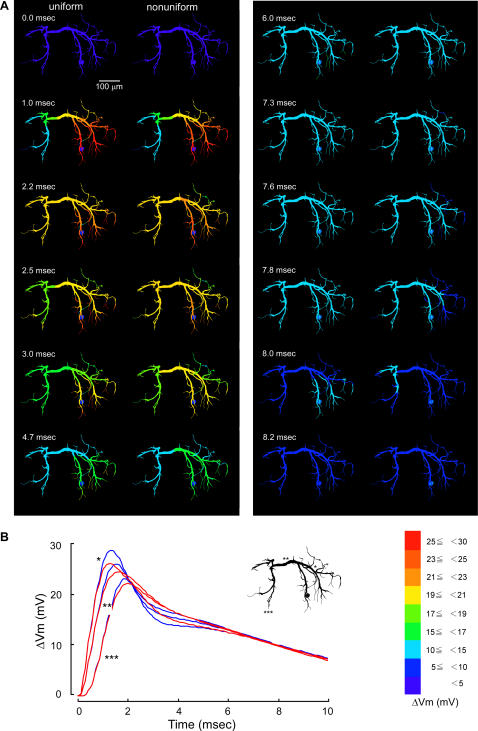
Effects of non-uniform distribution of active conductances in the pattern V on the dendritic spread of a compound synaptic potential. Colors and symbols are the same as in Fig. 3. The active conductances were distributed only on dendrites with the diameter of less than 4 µm except soma. The synaptic potential waveforms were similar to those calculated for the pattern II, different from those calculated for the uniform distribution not only on the side ipsilateral to the synaptic input, but also on the midline and on the opposite side.

The difference was more prominent when the active conductances were distributed in the patterns II, IV and V. Under the distribution pattern II, the compound synaptic potential decayed more slowly than under uniform distribution in the dendrites on the side opposite to the soma and the transverse segment on the midline (2.5–3.0 msec in Fig. 5A right column) whereas it decayed more rapidly later (>6.0 msec) in the fine dendritic terminals where active conductances were heavily distributed on the side ipsilateral to the soma. The depolarization due to synaptic activation is relatively large on the soma side so that the potassium conductances are effectively activated to steepen the falling phase of the response (1.0 msec in Fig. 5A) while the peak amplitude of the synaptic potential is smaller on the opposite side than on the soma side that the activation of potassium conductances are ineffective to make the falling phase slower in its time course although those conductances are selectively distributed on thin peripheral dendrites (7.0–7.9 msec in Fig. 5A). The time course of the voltage decay is illustrated in Fig. 5B for three representative regions in the dendrite, i.e., the transverse segment (**), a thick dendritic region (*) on the way from the input terminal to the segment, and an output terminal (***) in the dendrite on the side opposite to the synaptic input. The spatio-temporal distribution of the compound synaptic potential observed in the distribution pattern II (Fig. 5) was almost the same with that observed in the distribution pattern V (Fig. 6). Thus the compound synaptic potential decayed more slowly in the contralateral dendrites to the soma and the transverse segment on the midline (2.5–3.0 msec in Fig. 6A right column) whereas it decayed more rapidly later (>7.0 msec) in the fine dendritic terminals on the side ipsilateral to the soma (7.3–8.0 msec in Fig. 6A right) than the synaptic potential evoked in the uniform dendrites. In all three representative dendritic regions, the peak amplitude was smaller, the peak time shorter, and the depression in the decay phase was less prominent in the nonuniform distribution pattern V than in the uniform distribution (Fig. 6B).

**Figure 7:**
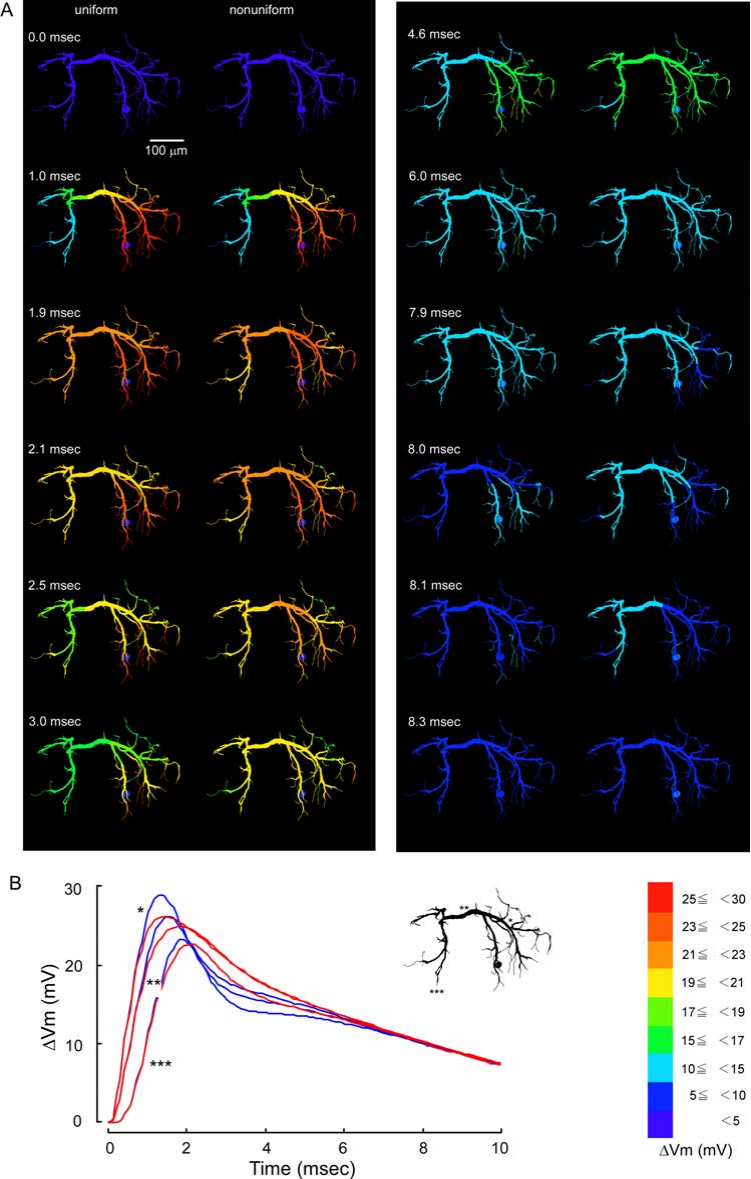
Effects of non-uniform distribution of active conductances in the pattern IV on the dendritic spread of a compound synaptic potential. Colors and symbols are the same as in Fig. 3. The active conductances were distributed only on dendrites with the diameter of less than 2 µm except soma is illustrated for twelve representative phases. This distribution pattern yielded the most different waveforms of synaptic potential among all five patterns currently examined.

**Figure 8:**
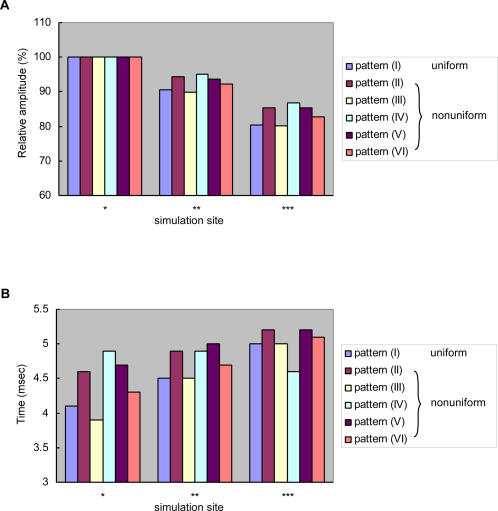
Peak amplitude (A) and half decay time (B) in the dendritic spread of a compound synaptic potential when active conductances are distributed uniformly (I) or nonuniformly in various patterns (II–VI). The peak attenuation is normalized to the peak value in a specific region on the dendrite ipsilateral to the soma. Single (*), double (**) and triple asterisks (***) indicate respectively the regions shown in Fig. 3B–[Fig pone-0002217-g004] [Fig pone-0002217-g005] [Fig pone-0002217-g006] 7B.

The distribution pattern IV was found to have the most significant effect on the spatio-temporal spread of the compound synaptic potential over dendrites. The synaptic potential spreads over the nonuniform dendrite to the contralateral processes less effectively (1.9 msec in Fig. 7A right column) and decays more slowly on both sides (2.1–8.1 msec in Fig. 7A right) than that spread over the uniform dendrite (Fig. 7A left). Temporal distributions of the synaptic potential at representative dendritic regions are shown in Fig. 7B when the active conductances were distributed only to dendrites having a diameter less than 2 µm (pattern IV). The peak time for the nonuniform distribution in this pattern was most deviated from that for the uniform distribution in the midline region (Fig. 7B **) unlike in other nonuniform distribution patterns for which the peak time difference was most remarkable (Figs. 4–6 *) in the dendritic regions on the side ipsilateral to the soma, i.e., the side of synaptic stimulation, while it was almost the same in other dendritic regions (Figs. 4–6 **, ***). The peak amplitude was most noticeably different between uniform and nonuniform distribution in some dendritic terminals receiving no synaptic input on the soma side (data not shown): it was larger by about 18% in the uniform than in the nonuniform distribution. This difference can be of physiological significance since the synaptic response of the interneuron to sensory stimulation at a certain intensity is invariable (see Materials and Methods). These findings, demonstrating the synaptic potential waveforms most deviated from those observed in physiological experiments and in calculation on the model with uniform distribution pattern of active conductances, suggested that the possibility of their nonuniform distribution in the pattern IV over the dendrite is the smallest in the LDS interneuron in vivo. Effects of nonuniform distribution patterns of active conductances on the synaptic potential waveform are summarized in Fig. 8 where the waveforms are normalized to the compound synaptic potential calculated for a specific region on the dendrite ipsilateral to the soma for each distribution pattern.

## Discussion

Dendrites of central nerve cells receive and process inputs from many excitatory and inhibitory synapses distributed over their surface. Integration of those input on dendrites is the fundamental basis of neuronal computation [23] and at the center of higher-order central nervous system functions [24]. Dendrites were first thought to be electrically passive so that the integration of synaptic input could be described adequately by a combination of linear cable theory and morphometric description of dendritic geometry [25]. It is true that passive membrane models provide a fundamental basic on which active membrane properties can be added to reflect actual membrane dynamics for understanding the spatio-temporal distribution of synaptic potentials over dendrites [1], [26], [27], but a growing amount of experimental data on a variety of voltage- and ligand-regulated conductances in a variety of central neurons [28]–[30] urges us to investigate into the functional significance of those conductances in each specific nerve cell in terms of its unique function in information processing and behavioral control. One important issue regarding the active dendrite is whether active conductances are distributed over dendrites uniformly or not [31]. In this study, different patterns of active conductance distribution over dendrites were shown to have potential effects on spatio-temporal spread of synaptic potentials from the input to output regions in an identified nonspiking interneuron of crayfish. Effective distribution patterns of active conductances and their functional significances are discussed below.

### Nonuniform distribution of potassium conductances over dendrites of the LDS interneuron

In a previous simulation based on the multicompartmental model of the LDS interneuron, we tuned the model by comparing voltage responses to intracellularly injected current pulses recorded in the transverse segment over the midline with those calculated in the compartment that corresponded to the transverse segment [17]. It was confirmed then that the recorded responses to current injection and stimulation of sensory afferents as well could be reproduced realistically by assuming that all three types of potassium conductances [15] were distributed uniformly over the cell except for the cell body. We did not examine systematically in the previous study, however, the effect of nonuniform distribution of active conductances over the cell on the voltage responses. The possibility was supported by the previous simulation that the dendrite is distributed by active conductances uniformly, but another possibility could not be excluded that the conductances were distributed nonuniformly over dendrites without affecting the temporal distribution of the voltage in the transverse segment. In the present study, we systematically changed the distribution pattern of active conductances over dendrites and examined its effect on the synaptic activity in various regions within the cell including the transverse segment to test the remaining possibility.

We tested in the present study six representative distribution patterns of active conductances including their uniform distribution over the entire dendrite. It has been reported in vertebrate central neurons that the cell body, adjacent to the spike initiating region of axon hillock playing a critical role in synaptic integration, is distributed by active conductances more or less densely than dendritic processes [7], [32], [33]. In invertebrate central neurons, however, the cell body is out of the pathway from the input to output regions within a cell [34], [35], the thick dendritic segment acting as the integrating site for synaptic input. Hence we distributed active conductances over dendrites of the LDS interneuron in various nonuniformity centering the transverse segment on the midline.

The results on unitary synaptic potential evoked at a dendritic terminal on the side ipsilateral to the soma (Fig. 2A) showed that the spatio-temporal spread of the potential remained unaffected whichever distribution pattern was implemented in the model including the uniform distribution over dendrites. In any distribution pattern, the peak amplitude was greatly reduced while the time course was expanded when the potential spread to thick (>8 µm) segments of ipsilateral dendrites and further to the transverse segment (Fig. 2A). It should be noted here that, regardless of how the active conductances were distributed, the temporal profile of the unitary synaptic potential at the dendritic terminal where the activated synapse was located was not affected at all by active conductances even when the membrane potential exceeded the activation level for three types of potassium conductances [15]. Even when the active conductances were exclusively distributed on fine dendrites including the synaptic site, the waveform of the potential at the site of its origination as well as other dendritic regions was the same as that when the conductances were distributed in other patterns (data not shown). The reason for this invariability is unknown, but one possibility would be that the amount of activated conductances was too small for affecting the voltage response due to synaptic action since only a fraction of the total membrane conductance measured in voltage-clamp experiments was assigned to the synaptic terminal compartment according to its membrane ratio to the whole active membrane.

The waveform of compound synaptic potentials as a result of spatial summation of unitary potentials that were evoked simultaneously at 94 synaptic terminals in the present simulation was found to be critically affected by the distribution pattern of active conductances. When they were distributed decreasingly from the thick transverse segment to distal branches (pattern III), the waveforms of compound synaptic potentials in representative three dendritic regions was almost similar to that calculated in the condition that they were distributed uniformly over the entire dendrite (Fig. 3B) although a little difference was observed at 2.0 msec and 7.9 msec after the onset of synaptic activity, i.e., in the decay phase of the synaptic potential (Fig. 3A). When the conductances were distributed only on dendrites having a diameter smaller than 8 µm, the waveform in the transverse segment (** in Fig. 4B) and the output region on the contralateral dendrite (***) was almost the same as that calculated for uniform distribution (blue traces in Fig. 4B). A little difference was also observed in the decay phase of the synaptic potential (2.0 and 7.8 msec in Fig. 4A). However, in the thick dendrite on the side ipsilateral to the cell body and the input synapse, the waveform in the distribution pattern VI was clearly different from that in the normal distribution (pattern I). These results, together with the fact that the recorded responses are realistically reproduced in the model with active conductances uniformly distributed over the entire dendrite [17], suggest that the distribution patterns III and VI cannot be excluded by experimental results.

When the active conductances were distributed increasingly from the thick transverse segment to distal branches (pattern II, Fig. 5B), only on dendrites having a diameter smaller than 2 µm (IV, Fig. 7B), or only on dendrites having a diameter smaller than 4 µm (V, Fig. 6B), the waveform of the compound synaptic potential in the transverse segment as wells as in other regions was significantly different from that calculated for uniform distribution. It is thus suggested that these nonuniform distribution patterns of active conductances are unlikely to be the case for the dendrite of the LDS interneuron. It is true that the recorded responses of the interneuron to stimulation of sensory afferents varied depending on the preparation [15] so that the comparison between recorded responses and calculated responses for the distribution patterns II, IV and V cannot be definitive. It should be pointed out, however, that although the recorded compound synaptic potential in response to afferent stimulation varied among preparation, the characteristic depression following the rapid decay from the peak was common to most variations that differed in the overall time course of the synaptic response. The absence of this depression therefore strongly suggests that the distribution patterns II, IV and V would be unlikely in the LDS interneuron.

The soma conductance, however assigned in addition to uniform distribution, was shown to have no significant effect on the compound synaptic potential as far as the stimulus intensity was kept maximal (Fig. 2C). Since the comparison of recorded and calculated responses in tuning the model was always done with the maximal response, we can ignore the difference observed in the medium-sized responses between passive and active soma membranes (Fig. 2C) to conclude that the any possible soma conductance is not functionally significant in the synaptic integration of the LDS interneuron.

### Potential significance of nonuniform distribution of active conductances in synaptic integration

**Figure 9:**
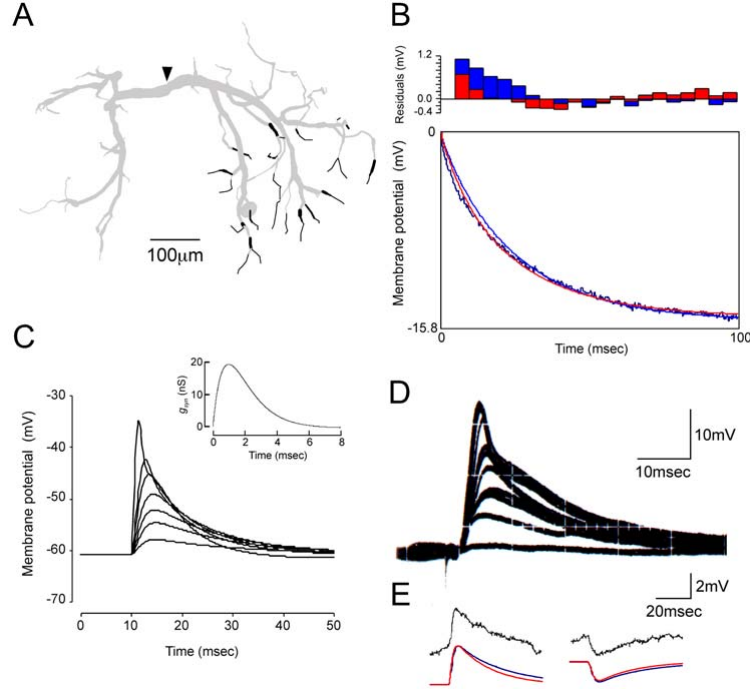
A multicompartmental model of the LDS interneuron. A: Representation of the interneuron by an assembly of 493 cylinders having different diameter and length values. The arrowhead indicates a compartment that corresponds to the site of electrode impalement into the interneuron during experiment. Those terminals shown in black color are those assumed to bear input synapses from the sensory afferents. B: Comparison of the voltage response to a step current injection (−1 nA) recorded in the region shown with an arrowhead in A with the calculated responses. Since a single electrode was used for both current injection and recording (see Materials and Methods), the arrowhead indicate the site of current injection as well. By changing the soma and dendritic membrane area, the voltage response could be made closer to the recorded response (blue and red lines). In this simulation, we used the soma and dendritic membrane area values that yielded the blue calculation. C: Calculation of compound synaptic responses to increasing stimulus intensity in the midline region shown by an arrowhead in A. The stimulus intensity was regulated by the number of activated synapses in the calculation: a larger number of activated synapses in simulation corresponded to a larger number of sensory nerve axons each firing a single action potential in the physiological experiment. The inset indicates the profile of synaptic conductance change used in this simulation for a single synaptic activation. D: Compound synaptic responses to synaptic stimulation recorded in the midline region of the LDS interneuron. Stimulus intensity was changed in seven steps from the minimal to the maximal. For each intensity, the stimulation was repeated several times. The synaptic response of the interneuron to sensory stimulation at a certain intensity is invariable. At the sixth step from the minimal, the stimulation was carried out only once. This was because we were trying to find the maximal intensity. E: Unitary depolarizing (left) and hyperpolarizing (right) synaptic potentials recorded (upper) and calculated (lower) in the midline region. The blue line shows the calculated response in the absence of active conductances while the red one calculated in their presence.

The results of current simulation suggest that, judging from the calculated waveform of compound synaptic potentials, the active potassium conductances can be distributed either uniformly or nonuniformly in the patterns III or VI over the dendrites of the LDS interneuron. In the pattern III when the conductances were distributed decreasingly from the thick transverse segment to distal branches, the synaptic response was always the same with that under the condition of uniform distribution (Figs. 3B, 9). The nonuniform distribution of conductances in this pattern has therefore no functional significance in the spatio-temporal distribution of synaptic potentials over dendrites and hence in synaptic integration. In contrast, when the active conductances were distributed in the pattern VI, i.e., when they were distributed only on dendrites having a diameter smaller than 8 µm, the synaptic potential in the output region on the contralateral dendrite was almost the same as that under the condition of uniform distribution (Figs 4B, 9). In the ipsilateral dendrite, however, it was significantly different from that calculated when the conductances were distributed uniformly (Fig. 4B). In this case, the nonuniform distribution will not affect the synaptic activity at the synaptic terminal and its spread over the transverse segment to contralateral dendrites, but will affect the synaptic output from the ipsilateral dendrite on the soma side. The LDS interneuron is a nonspiking interneuron that generates a spike nowhere in the cell exerting synaptic output from synapses on the dendrite [12]. Kondoh and Hisada [22] reported that in the LDS interneuron most output synapses are located in the dendrite contralateral to the soma, but yet a small number of them in the ipsilateral dendrite. Compared with uniform distribution of active conductances, their pattern VI distribution would weaken the synaptic output from the ipsilateral output synapse whose target cell remains unknown. Depending on the possible roles of the postsynaptic cell, this suppression of ipsilateral synaptic output from the LDS interneuron as an inhibitory cell [12], [13] could potentially function in, e.g., contrasting the inhibitory effect on both sides or adjusting the recurrent feedback pathway.

Nonuniform distribution of voltage-regulated conductances has been reported in a variety of central nerve cells in the vertebrate brain. In the hippocampal CA1 pyramidal cell, the voltage-regulated potassium conductance is distributed increasingly from the soma to peripheral dendrites [7] as the pattern II in the present study whereas the voltage-regulated calcium conductance is distributed discontinuously in patches on the dendrites of cerebellar Purkinje cells [3]. The distribution of potassium conductances in pattern VI can be regarded as a continuous increase on a nonlinear scale instead of the linear one as in the patterns II and III, although the conductances are actually distributed in patches of peripheral dendrites (Fig. 1E). When we regard the transverse segment as the functional equivalent of the cell body in vertebrate central neurons, the nonuniform distribution pattern of potassium conductance on the LDS interneuron dendrite appears to be common to that reported in many vertebrate neurons where active conductances are distributed more densely in more peripheral dendrites from the soma [7], [36]. The physiological significance of this nonuniform distribution pattern should vary depending on the functional role of each neuron in behavioral control and information processing. Further analysis is needed on the distribution of potassium conductances in the LDS interneuron since the conclusion in this study has been reached by the process of elimination: the possibility that they are nonuniformly distributed in the pattern III or VI could not be excluded while other possibilities of nonuniform distribution pattern (II, IV, V) were judged to be unlikely. Positive experimental evidence for the pattern III or VI distribution of potassium conductances could be approached either electrophysiologically by focal patch recording or anatomically by immunocytochemical procedures.

## Materials and Methods

### Experimental data

Electrophysiological and morphological data for constructing the present multicompartmental model were all obtained in our previous experimental studies. Here we concisely recapitulate experimental procedures and obtained results that are relevant to the present study. Full details on electrophysiological experiments are provided in Takashima and Takahata [15] and Takashima et al. [17] whereas those on the three-dimensional morphometry can be found in Hikosaka et al. [37].

#### ELECTROPHYSIOLOGY

The abdominal nerve cord including the terminal abdominal ganglion of adult crayfish, *Procambarus clarkii* Girard, was isolated from the rest of the body in crayfish saline. The LDS interneuron was impaled at the transverse segment on or near the midline of the ganglion from the dorsal side by a glass microelectrode filled with 3 M KCl having DC resistance of 10–15 MΩ measured in the saline. The electrode was coupled to a current/voltage clamp amplifier (Axoclamp 2B; Axon Instruments) so that it could be electronically switched to recording and current injection circuit in an alternate way. After optimal capacity compensation, the switched current- and voltage-clamp recordings (30% duty cycle for current injection) could be made at the rate between 4 and 5 kHz. Clamp gain was dependent on the electrode condition and set in every experiment so that oscillation just began to be seen on termination of a depolarizing voltage pulse. It was between 1.5 and 3.0 nA/mV in this study. In the voltage-clamp mode, the cell was held at its resting potential level (65.0±5.7 mV). The output bandwidth of the amplifier was generally set at 1 kHz. Voltage and current signals were lead from the amplifier to a digital oscilloscope (Tektronix 2232) for visual observation and to a DAT data recorder (TEAC RD-135T; band width DC-10 kHz) for later replay on the oscilloscope. Current and voltage recordings were obtained by averaging over 4 to 10 repeated trials on a digital oscilloscope (Tektronix 2232). Digitized data (8 bit vertical resolution for a single sweep and 10 bit for averaging sweep) were sent to an Apple Macintosh computer equipped with a GPIB interface (GW Instruments) for data analyses.

For isolating the voltage-dependent membrane conductances of the LDS interneuron, we used tetraethylammonium chloride (Sigma T2265) and 4-aminopyridine (A0152). They were applied to the LDS interneuron in the ganglion by perfusing the experimental chamber with the drug dissolved in the saline. Details of the perfusion method is found in a separate paper [14]. The recorded current for each clamp voltage after drug application was then subtracted from the current for the corresponding clamp voltage before drug application to yield the time- and voltage-dependent profile of the conductance that was specifically affected by the tested drug. We could finally isolate a TEA-sensitive sustained conductance (*gs*) and two types of transient conductances, one (*gt1*) sensitive to TEA and another (*gt2*) sensitive to 4-AP [15].

#### 3-D MORPHOMETRY

In the morphological examination, the LDS interneuron was stained with Lucifer yellow CH, fixed in 10% formalin, dehydrated through ascending ethanol series, and cleared in methylsalicylate. The ganglion was then optically sectioned using a confocal laser scanning microscope system (Molecular Dynamics, Sarastro 2000) including an Argon-ion laser, a Nikon Optiphoto II epifluorescence microscope and a Silicon Graphics Iris Indigo XS24 workstation. The preparation was always set horizontally with the dorsal side up on the microscope stage. With an objective lens of ×10 (NA = 0.45), each section was resolved into either 512×512 pixels with the size of 2×2 µm or 1024×1024 pixels of 1×1 µm in 256 gray scales. The scanning plane was then changed by 1 or 2 µm in the Z-axis direction corresponding to the dorso-ventral axis of the animal body, using a stepping motor attached to the fine control of the microscope stage.

Sectioning the whole cell including the cell body and all dendritic branches resulted in a series of 120 to 223 sections with the stepsize of 2 µm. They were stored in a magneto-optical disk for later analyses. We measured the diameter and length of each dendritic branch on the serial sections by ImageSpace (version 3.0; Molecular Dynamics, Sunnyvale, CA), a software for image/volume analysis which works with the Sarastro 2000 system. Each dendritic branch was represented by one cylinder or a connected sequence of cylinders each with specific diameter and length values. The whole LDS interneuron consisted of 418–645 cylinders or compartments. The length of each cylinder was calculated trigonometrically from the three-dimensional coordinates of the centers of its both ends. The diameter and length of dendrites in vivo were obtained by multiplying the measure with the shrinkage factor reported in a previous study [37]. Values used for constructing the present model are all those after the shrinkage compensation. The coordinates for each process were stored together with its diameter in a database running on a personal computer for 3-dimensional reconstruction.

### Model construction

The LDS interneuron used in the current modeling was represented by an assembly of 493 cylindrical compartments each having specific diameter and length values (Fig. 9A; [38], [39], [40]). Each compartment was connected with its neighbors with axial resistances. The electrotonic length of a single compartment was chosen to be less than 0.1λ, where λ is the length constant, so that each compartment could be regarded as isopotential. The axial resistance and λ values were obtained using the result of 3-D morphometry on the dendritic diameter as well as the result of electrophysiological experiment on the membrane time constant τ_m_. The axoplasm resistivity and the membrane capacitance per unit area were assumed to be 0.06 kΩcm [41] and 1 µF/cm^2^ [23], [42] respectively. Experimental procedures for obtaining the membrane time constant from the voltage response to step current injection are described elsewhere in detail [43]. In the cell that was used to construct the multicompartment model for the present study, the experimentally obtained value for τ_m_ was 25.8 msec as measured in the thick transverse segment over the midline. The membrane resistance per unit area was assumed to be uniform over the whole dendrite. Also incorporated into the present model were three kinds of voltage-dependent outward conductances mentioned above as well as a leak conductance (*g_leak_*). Details on numerical reconstruction of these conductances based on Hodgkin and Huxley [44] were described in a previous paper [15]. Experimental analyses have suggested that all of voltage-dependent currents are carried by potassium ions.

The whole ionic current flowing through the membrane including the leak current is expressed as
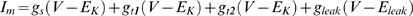
(1)in which *E_K_* and *E_leak_* are the equilibrium potentials for outward and leak current respectively. *E_K_* was −70.0 mV according to the experimental data. *E_leak_* was assumed to be the same as the resting potential. The voltage- and time-dependent parameters for the three potassium conductances (*gs*, *gt1* and *gt2*) were based on our previous experimental data [15]. The membrane potential of the *i*th compartment (*V_i_*) as a function of time was therefore obtained by numerically solving a set of first-order ordinary differential equations
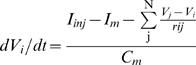
(2)in which *C_m_* represents the total membrane capacitance calculated from the membrane area, *I_inj_* the current injected intracellularly, *r* the axial resistances connecting the compartment with its N neighbors, and *I_m_* given by eq. 1.

The voltage-dependent membrane conductances were numerically reconstructed according to Hodgkin and Huxley [44] by fitting experimental results to the general form of equation:
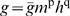
where *g* is the membrane conductance and *g̅* is the maximal membrane conductance for the potassium ion. The values for g and *g̅* were obtained experimentally [15]. *m* and *h* are voltage- and time-dependent gating variables for activation and inactivation respectively, ranging between 0 and 1. The gating variable dynamics were modeled as the solution to the equation
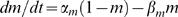
in which α*_m_* and β*_m_* are voltage-dependent rate constants for transition of the gate to permissive and non-permissive state respectively [45] based on the assumption that the transition obeys first-order kinetics. α*_m_* and β*_m_* in the present study were described by either exponential or sigmoid function of voltage in the following general forms:
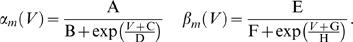



Values for parameters A - H were determined on the basis of our experimental data [15] and summarized in a table [17].

### Model verification

The constructed model was verified by comparing the recorded response to a step hyperpolarizing current (1 nA) injected into the thick transverse segment with the calculated response of the model to the same current injection into a compartment that corresponded to the transverse segment (Fig. 9B). In constructing the model, the results of three-dimensional morphometry on the compartmental diameter were multiplied with 1.2 to compensate for the shrinkage due to fixation and clearing procedures that followed fixation [37]. It was found, however, even after this shrinkage compensation, the calculated response of the model was significantly larger than the recorded one, suggesting that the total membrane area in the morphological measurement was underestimated. This underestimation appears to be partly due to the current procedure that each dendritic branch was approximated by one or more cylindrical compartments. It was also likely that those processes with diameters less than 1 µm might have eluded the present measurement that was limited by the spatial resolution of the confocal laser scanning microscope system [16], [37]. In addition, electronmicroscopic studies have revealed that the membrane of the soma and dendrites is not smooth but generally shows irregular profiles [22], [46], [47], [48]. In the present model, the model was tuned by changing the soma and dendritic membrane area. They were finally assumed to be 5 times and 1.27 times larger than the measurement respectively without a change in the axial resistance so that the recorded and calculated responses were made comparable (Fig. 9B).

### Simulation of Synaptic Activities

#### SYNAPTIC INPUT

A previous study showed that the LDS interneuron receives excitatory synaptic input on the side where its cell body is located [12]. Simultaneous intracellular staining of the LDS interneuron and its presynaptic mechanosensory afferents in the same preparation [17] has suggested that the afferent fibers make synaptic contacts with the interneuron at its distal dendrites. This observation is consistent with the result of electronmicroscopic study that input synapses of the LDS interneuron are mostly distributed on fine branches rather than on thick ones [22]. In this study, the branch terminals on the soma side were assumed to bear synaptic sites receiving the afferent input (Fig. 9A). Synaptic responses of the model cell were calculated by adding a term representing the synaptic current in the form of *gsyn*(*Vi* – *Esyn*) to the equation for *I_m_* (eq. 1) of the terminal compartment. *gsyn* is the synaptic conductance and *Esyn* the reversal potential that is assumed to be 0 mV for the excitatory input and −70 mV for the inhibitory input. Since the synaptic connection of the LDS interneuron with afferents was limited to within a small area in the ganglion (Fig. 9A), we assumed that the synaptic activation of dendritic terminals occurs simultaneously, the number of activated terminals representing the input strength. Thus stronger synaptic input was embodied by a larger number of activated terminals in the present model (Fig. 9C). *gsyn* was assumed to follow the alpha function (inset to Fig. 9C) with the peak time of 1.0 msec. The number of compartments bearing the input synapse was adjusted to be 94 in this study so that the compound synaptic responses calculated in the midline-region compartment for multiple inputs was of comparable magnitude with the actual synaptic potentials recorded in physiological experiments (Fig. 9D). The maximal synaptic conductance in a unitary synaptic input on each terminal was adjusted so that the calculated unitary synaptic potential in the midline region was comparable with the recorded unitary synaptic potentials (Fig. 9E).

#### NUMERICAL INTEGRATION

Simulations were performed with GENESIS [49] on a Pentium IV-class PC by solving the simultaneous equations (eq. 2) numerically. For integration, we used the Hines algorithm [50] available in GENESIS version 2.0 [51]. Simulations were run with a time step of 0.02 msec. Initial control simulations using 0.01 msec showed that the 0.02 msec step produced numerically accurate results. For the model presented here, 200 msec of the LDS cell activity could be simulated in 5 min. Several hundred simulations were run, mostly to explore appropriate parameters.

### Distribution of active conductances

The LDS interneuron is endowed with three kinds of voltage-regulated potassium conductances that differ from each other in the chemical drug sensitivity and the voltage and time dependent activation/inactivation kinetics [15]. In this study, we assumed that these three types of conductances were always distributed in the same way over dendrites. Thus we examined the following six situations, taking into consideration the known distribution patterns of potassium conductances over dendrites of vertebrate neurons including neocortical and hippocampal pyramidal cells, cerebellar Purkinje cells and mitral cells of the olfactory cortex [31], [36], in which the three types of conductances as a whole are differently distributed over the dendrites of the LDS interneuron while the total membrane conductances of each type were kept constant over the whole dendrite. It should be noted here that in invertebrate neurons the thick dendritic segment, rather than the cell body, serves as the integrating site for synaptic inputs [34], [35]. The distribution of active conductances in the present study, therefore, reflected this situation.

- Conductances are uniformly distributed over the interneuron.
- Distributed increasingly from the thick transverse segment to distal branches (Fig. 1A).
- Distributed decreasingly from the thick transverse segment to distal branches (Fig. 1B).
- Distributed only on dendrites having a diameter smaller than 2 µm (Fig. 1C).
- Distributed only on dendrites having a diameter smaller than 4 µm (Fig. 1D).
- Distributed only on dendrites having a diameter smaller than 8 µm (Fig. 1E).
  In addition, to investigate the effect of cell body as an electrical load to the cell on the synaptic integration in the interneuron, we assumed the following three conditions.
- The cell body is distributed by additional 10% of the whole active conductances that are uniformly over dendrites.
- Distributed by additional 20% of the whole active conductances.
- Distributed by additional 40% of the whole active conductances.
